# False alarm on a malaria “outbreak” linked to inconsistencies in malaria diagnostic supply: a call to strengthen supply chain management: Sierra Leone, May–July 2023

**DOI:** 10.1186/s12936-025-05312-x

**Published:** 2025-03-26

**Authors:** Timothy N. DeVita, Aminata B. Kabia, James A. M. Khobi, Mugagga Malimbo, Samba Kamara, Bridget Magoba, Gebrekrstos N. Gebru, Anna H. Jammeh, John A. Painter, Thomas K. Ansumana, Musa Sillah-Kanu, David C. Schnabel

**Affiliations:** 1 Malaria Branch, U.S. Centers for Disease Control and Prevention, Atlanta, GA USA; 2 Epidemic Intelligence Service, U.S. Centers for Disease Control and Prevention, Atlanta, GA USA; 3 African Field Epidemiology Network, Freetown, Sierra Leone; 4 Sierra Leone Field Epidemiology Training Program, Freetown, Sierra Leone; 5 World Health Organization, Freetown, Sierra Leone; 6 United States President’s Malaria Initiative, United States Agency for International Development, Freetown, Sierra Leone; 7 United States President’s Malaria Initiative, Centers for Disease Control and Prevention, Atlanta, GA USA; 8 Ministry of Health, Freetown, Sierra Leone; 9 United States President’s Malaria Initiative, Centers for Disease Control and Prevention, Freetown, Sierra Leone

## Abstract

**Background:**

Malaria transmission in Sierra Leone is intense and perennial, accounting for 40% of clinical consultations. Medical workers diagnose suspected malaria cases using rapid diagnostic tests (RDT) and microscopy, with facility-level results reported to the Health Management Information System (HMIS) as monthly aggregates. Commodity stocks are reported to the Logistics Management Information System (LMIS). Partners investigated a striking increase in confirmed malaria during May–July 2023 in Sierra Leone, peaking in June to 46% above the June 2018–2022 mean.

**Methods:**

The team first analysed national, district, and facility HMIS/LMIS data for RDT stocks, testing rates, and confirmed cases during January 2018–October 2023. Epidemic thresholds, defined as case counts two standard deviations (σ) above the previous 5 years’ monthly mean, were assessed. Then four facilities in two districts were visited to interview staff. Lastly, the team reanalysed LMIS RDT stock data for all facilities in Sierra Leone using R to categorize their stock status by month.

**Results:**

National epidemic thresholds were surpassed in May (2.56σ) and June (4.81σ) 2023. Twelve of sixteen districts surpassed epidemic thresholds during May–June. Investigation revealed inconsistent RDT distribution to facilities over time. National RDT distribution spiked in May 2023, when 551,888 RDT test kits were delivered. This was substantially larger than the 2019–2022 mean for May (53,121, 1,000% increase) and all months (126,866, 435% increase). Subsequently in June 2023, 386,343 tests were performed, 36% higher than the June 2018–2022 mean (285,123). Staff at all four visited facilities reported recurrent RDT stockouts. The proportion of facilities in Sierra Leone reporting positive RDT stocks at both the start and end of the month increased from 14% in April to 74% in June. 51% of facilities began May with RDT stockout and received RDTs that month.

**Conclusions:**

The 2023 spike in confirmed malaria was likely related to increased testing following an unusually large distribution of RDTs. Fluctuations in RDT availability impede the ability to recognize true case variations. Sierra Leone and its partners can strengthen supply chain logistics and health commodity stock tracking to ensure a consistent supply of RDTs and improve interpretation of surveillance data.

## Background

Malaria presents one of the largest public health challenges globally, with 85 endemic countries, 249 million cases annually, and 608,000 deaths in 2022 [1]. Africa is disproportionately impacted, accounting for 94% of malaria cases. In Sierra Leone, malaria transmission is intense and perennial, with increases in April and October due to the beginning and end of rainy season. [2, 3]. The country’s entire 8.1 million population is at risk for the disease [4, 5]. In Sierra Leone, malaria had an incidence of 329.83 per 1,000 population at risk and prevalence of 22% by microscopy among children under 5 years of age in 2021 [5–7]. The parasite presents a formidable challenge to Sierra Leone’s healthcare system, accounting for 40% of the overall disease burden among outpatient visits, and approximately 1,000 fatalities yearly among children less than five years [8]. The pervasive health issue is further complicated by recent events, including the country’s civil war (1991–2002), a regional Ebola virus outbreak (2014–16), and the 2020 COVID-19 pandemic, which stretched the country’s limited healthcare resources [4, 8]. Considering this context, effective and efficient malaria diagnosis, case management, and surveillance in Sierra Leone is paramount to targeting limited resources and achieving maximal impact.

In Sierra Leone, national guidelines direct medical workers to test febrile patients with histidine protein-2 (HRP2) based rapid diagnostic tests (RDT) or microscopy to confirm malaria infection prior to treatment [9]. Staff record confirmed positive and confirmed negative malaria cases into each health facility’s paper register. On a monthly basis, each facility aggregates cases by sex, age (< 5 years, 5–14 years, >  = 15 years), type of confirmation test (microscopy vs RDT), and where the test is performed (community or facility). The aggregations are reported using a paper summary form and entered into the Health Management Information System (HMIS) using the District Health Information System 2 (DHIS2) platform [10]. Similarly, health commodity stock data are entered to the Logistics Management Information System (LMIS). Once entered, the data are available for analysis at the facility, chiefdom, district, and national levels.

From May to July 2023, HMIS data indicated a striking increase in confirmed malaria cases in Sierra Leone. Monthly confirmed cases nationally rose to a peak of 273,835 in June 2023, a 46% increase from the mean of the five previous Junes (2018–2022), 187,366 confirmed cases. Partners responded to this upsurge in confirmed malaria cases with an investigation to determine its geographic spread, duration, impacted demographics, and inciting factors.

## Methods

This project underwent human subjects review and was approved as a sub activity under the CDC protocol “Use of Routinely Collected Health Management Information System Data and other Routinely Collected Program Data in Support of the President’s Malaria Initiative (PMI).” The investigation comprised an analysis of malaria testing and confirmed case rates, site visits, and analysis of health commodity stocks. Data were downloaded from Sierra Leone’s HMIS and LMIS databases using DHIS2 version v2.31.9 in both November 2023 and March 2024 as additional data became available.

### Malaria testing and confirmed case data investigation

The following data elements were downloaded from HMIS on national and district levels for both RDT and microscopy: fever case tested for malaria—positive, fever case tested for malaria – negative. The following data elements were similarly downloaded, but only for RDT: fever case in community tested for malaria—positive, and fever case in community tested for malaria—negative. January–November 2023 were analysed due to data availability, with particular attention to the three peak months of May–July. To control for seasonal transmission variations, 2023 data were compared to the same month’s averages from the prior five years, 2018–2022.

First, monthly malaria testing rates were calculated, including all diagnostic methods (microscopy and RDT) and sites of testing (facility and community). The team conducted a sub-analysis on testing rates by site of test. Similar sub-analysis of malaria test by diagnostic method was not pursued because microscopy capacity in the country had increased consistently across the preceding five years, a 2400% increase since 2018, making year-to-year comparison uninterpretable. Test positivity rate (TPR) was also calculated by dividing the number malaria RDTs performed at facilities that were positive by the total number of RDTs performed at facilities. Microscopy and RDTs performed by community health workers were not included in TPR calculation because the number of negative results were frequently unreported for these data elements.

Confirmed malaria was further analysed by age group (< 5 years, 5–14 years, ≥ 15 years) and Sierra Leonean district. Number of confirmed cases were analysed to determine if they met epidemic thresholds, defined as case counts two standard deviations (σ) higher than the monthly mean calculated from the previous 5 years’ data [11, 12].

### Site visit investigation

The team conducted site visits to four facilities in two districts, Bo and Moyamba. The two districts were selected for both epidemiologic and logistical factors. Epidemiologically, the facilities all had pronounced increases in confirmed cases that were concurrent with national and district surveillance data. However, on the district level, Moyamba had a more pronounced increase in cases than Bo, its neighbouring district. Bo’s cases peaked in May 2023, whereas Moyamba’s cases peaked in June along with most districts. Logistically, Bo and Moyamba are along a major roadway from the capital, Freetown, enabling more facilities to be visited during the team’s limited time.

For each facility, the following data elements were downloaded from HMIS: fever case tested for malaria—positive, fever case tested for malaria—negative, fever case in community tested for malaria—positive, and fever case in community tested for malaria—negative. Again, 2023 data were compared to the same month’s averages from the prior five years to control for seasonal variations. During site visits, the team conducted key informant interviews with district and facility-level staff to identify root causes of the increase in reported cases. Interviews were semi-structured with open-ended questions. Confirmed case numbers were compared between health facility registers, paper HMIS summary forms, and electronically entered HMIS data in DHIS2 to determine if there were data aggregation or data entry errors. The team also examined RDT stock data reported to LMIS by each facility.

### Health commodity stock data investigation

Due to reports of RDT stockouts during site visits, LMIS data were used to investigate RDT stocks on national, district, and facility levels. The following malaria RDT supply chain data elements, which facilities report monthly, were downloaded from DHIS2: opening balance, quantity received, quantity dispensed, closing balance, and reported stockout. Data from 2019 to 2023 were utilized because LMIS stock data in DHIS2 commenced in 2019.

The team first attempted to quantify regularity of facility participation in LMIS stock data. Using the summarise function of the dplyr package on R data analysis software, the team calculated the number of months in a twelve-month period (October 2022–September 2023) that each facility reported stock data to HMIS [13, 14]. Facilities that responded to any one of three data elements, “opening balance,” “quantity received,” or “stockout,” were deemed to have an “adequate response” in a given month.

Next, routine LMIS data was examined as reported, without reanalysis. On the national level, the team examined the number of RDT test kits distributed and number of facilities reporting stockout of RDT each month.

Lastly, because routine LMIS data as reported revealed inconsistencies across data elements, the team used logical criteria to gain a better understanding of individual facility stock statuses by month. Using the dplyr package and case_when function on R, each facility was designated one of thirteen stock statuses for each month from November 2022–September 2023 [13, 14]. The five main categories were “Stocked throughout month,” defined as facilities that began and ended a given month with RDT stocks; “Reported Stockout,” defined as facilities that reported a stockout at the end of a given month; “Likely Stockout,” defined as (1) facilities that used an amount of RDTs equal to the sum of their opening balance and quantity received, (2) had a zero closing balance for that month and a zero opening balance the subsequent month, and (3) did not report a stockout; “Newly Restocked After Stockout”, defined as facilities that were stocked out at the beginning of the month, received RDTs, and were not stocked out at the end of the month; and lastly “Unknown/Not Reported,” defined as not reporting anything in a given month or not meeting the logical criteria for other stock statuses. The lengths of reported and likely stockouts were delineated as new (< 1 month), > 1 month, or > 2 months, as possible.

## Results

### Confirmed case and testing data analysis

**Fig. 1:**
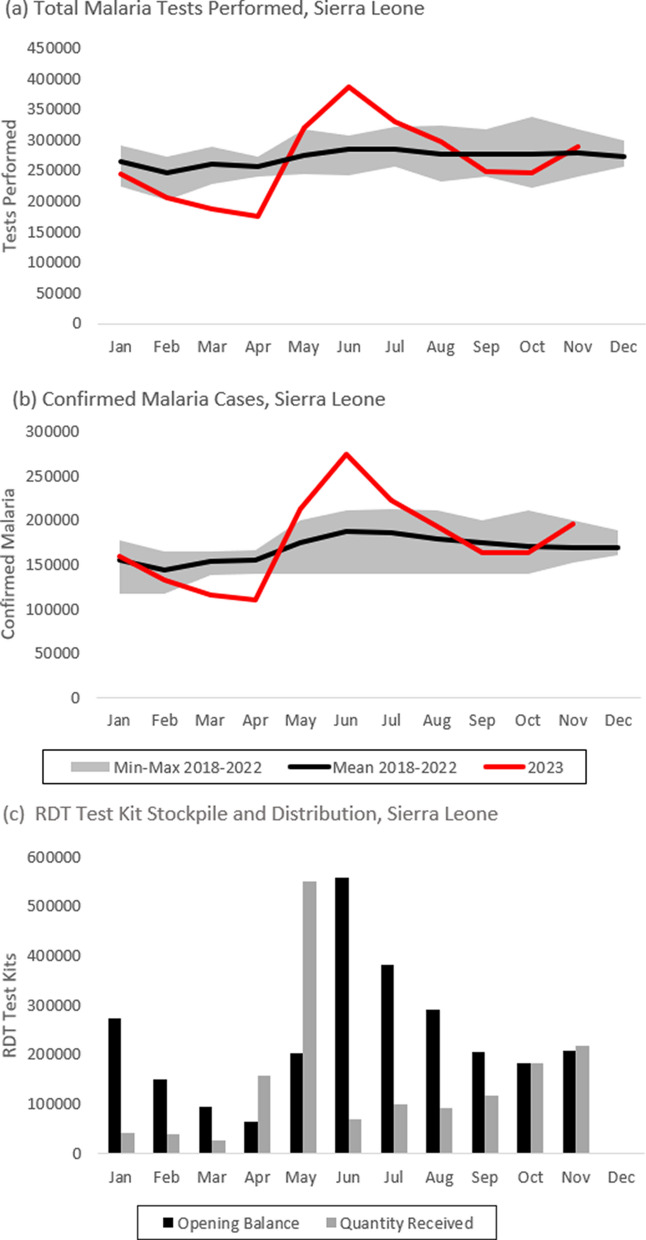
Confirmed Malaria, Testing, and RDT Stockpile Distribution, Sierra Leone, 2023

During the period under investigation, there was a substantial increase in both malaria testing and confirmed malaria reported to HMIS. The total malaria tests performed monthly in Sierra Leone from May to July 2023 surpassed all other months in the six years analysed. Testing rates peaked in June (Fig. 1a), contemporaneously with the spike in cases. Total tests performed in June 2023 increased 36% (386,343 tests, 4.4 σ) from the June 2018–2022 mean of 285,123. March and April 2023, which immediately preceded the upsurge in cases beginning in May 2023, were the two months with the lowest number of tests performed in the six years analysed (− 3.67 σ and − 5.98 σ). Tests performed by community health workers increased 283% to 92,817 tests from the June 2018–2022 mean of 32,783. Though malaria testing and confirmed cases increased dramatically, the test positivity rate of RDTs performed at facilities in June 2023 (72.0%) had modest increase from the June 2018–2022 mean (65.2%). However, there were consistently more facilities that reported positive malaria tests than facilities that reported negative malaria tests. Of the facilities that reported positive malaria tests, 8.5% did not report numbers of negative tests. This made test positivity rate calculation a consistently inaccurate estimation.

Monthly confirmed malaria cases in Sierra Leone from May to July 2023 surpassed all other months of the six years analysed, peaking in June 2023 (Fig. 1b). The epidemic threshold, two standard deviations above the 2018–2022 mean, was surpassed in May (2.56 σ) and June (4.81 σ) [8, 9]. July 2023 was just below the threshold (1.9 σ), Confirmed cases in June increased to 273,835 from the June 2018–2022 mean of 187,366 (46%). As with the trend of confirmed cases, the two months of March and April 2023 had the lowest number of confirmed cases of the five years analysed (− 4.2 σ and − 6.8 σ). The increase in confirmed malaria cases in June 2023 was experienced by all aggregated age groups (< 5 years, 5–14 years, ≥ 15 years), with the largest increase among children ages 5–14 (Table 1).

**Table 1:** Confirmed Malaria Cases by Age, Sierra Leone, June 2018–2023

Age	June 2018–2022 mean	June 2023	Percent increase
< 5 years	102,283	129,574	27
5–4 years	29,577	55,230	86
> = 15 years	89,031	55,506	60

The confirmed case distribution was also diffuse geographically. All 16 districts experienced a peak in cases, with most peaking in June (Table 2). Western Area Urban and Western Area Rural Districts, which comprise the capital Freetown and surrounding areas, and Bo District peaked earlier in May. Kailahun District, on the opposite side of the country to Freetown, peaked later in July. Twelve of sixteen districts met epidemic thresholds, with particularly large increases in the Falaba and Kono districts.

**Table 2:**
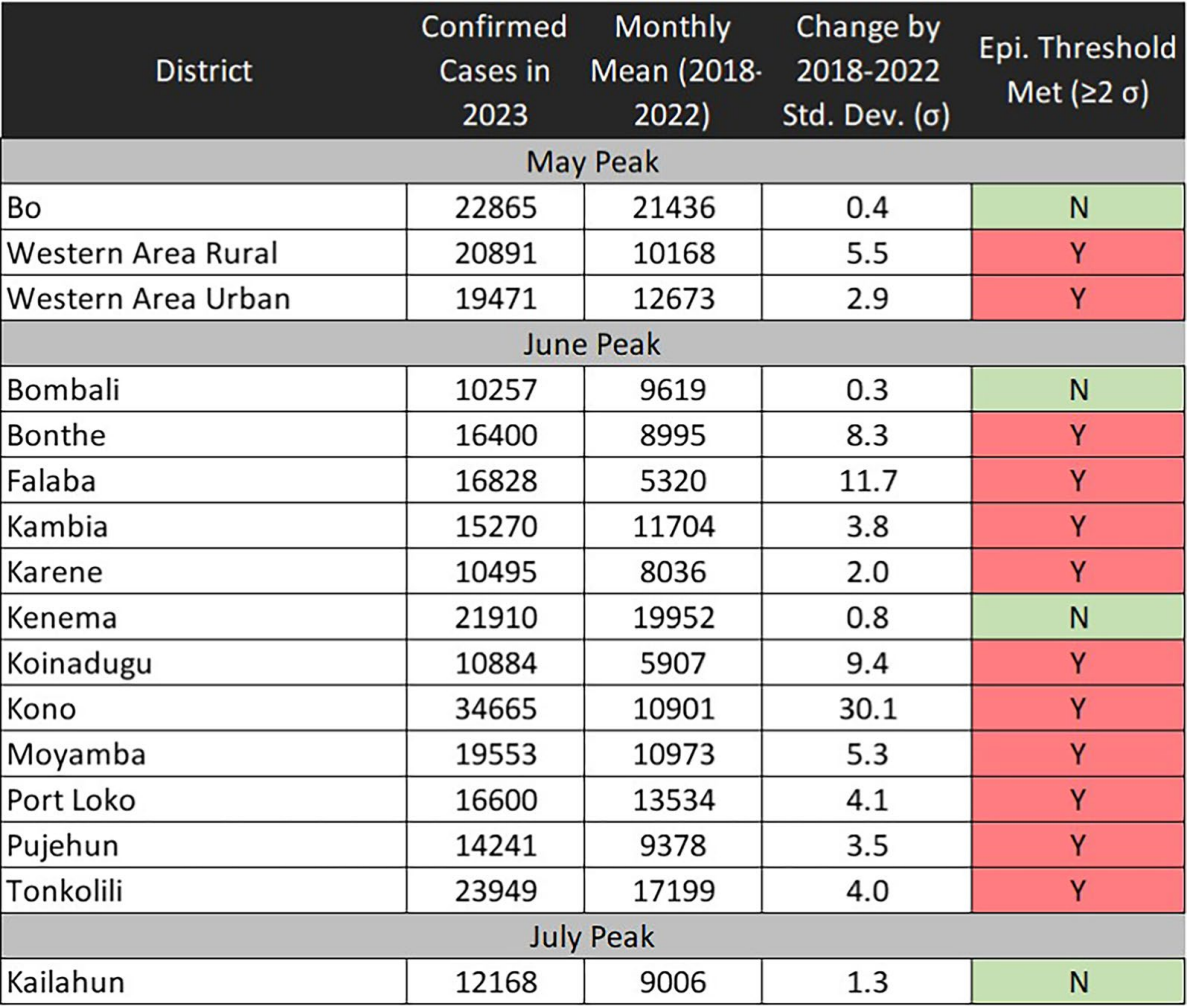
2023 Peak in Confirmed Malaria Cases by District, Sierra Leone, May–July 2023

### Site visit analysis

The four visited facilities had notably large increases in confirmed malaria cases, far surpassing epidemic thresholds and national trends. The two facilities in Bo, which peaked in May 2023, had combined 1525 cases versus the May 2018–2022 mean of 400 cases (381% increase, 11.3 σ). The Moyamba facilities peaked in June 2023 and had 1168 cases versus the June 2018–2022 mean of 315 (370% increase, 10 σ). The number of confirmed malaria cases was consistent across disaggregated facility registers, aggregated paper HMIS summary forms, and aggregated data reported to DHIS2 in three of four facilities. One facility had a large inconsistency between their aggregated HMIS summary form and aggregated data in DHIS2, indicative of a data entry error. Staff universally communicated recurrent RDT stockouts, with all facilities reporting a greater than 1-month RDT stockout immediately preceding their spike in cases.

Despite staff indicating that stockouts are a frequent problem at their facilities, LMIS analysis showed that the same facilities’ staff insistently and/or inaccurately reported RDT stock data. In particular, the data element “stockout” was inconsistently reported—two of the four visited facilities only reported the presence of a stockout the month when stockout began and not in the subsequent months despite still not receiving any new stocks.

### Health commodity stock data analysis

**Fig. 2:**
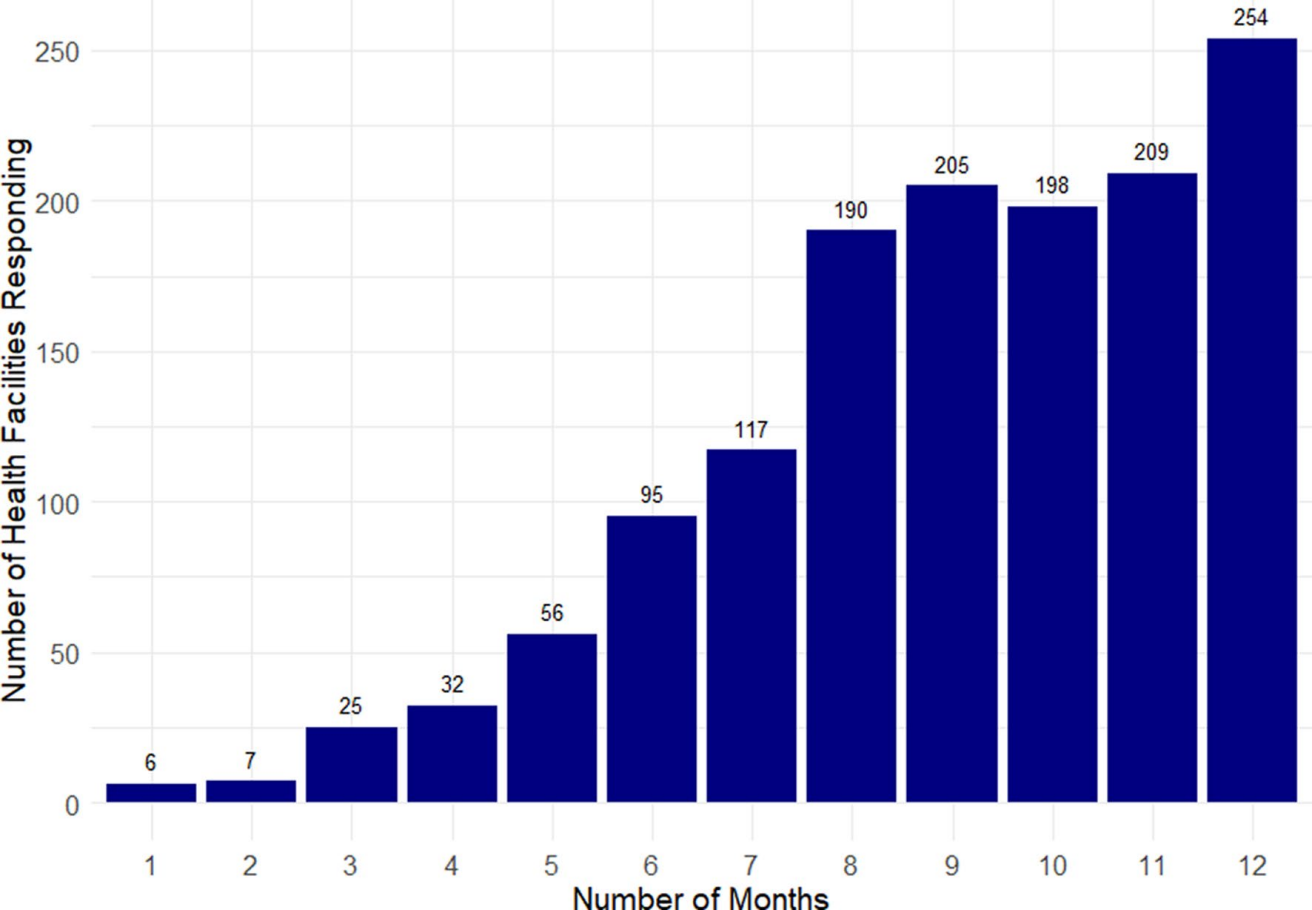
Consistency of Facility Participation in RDT Stock Reporting, Sierra Leone, October 2022-September 2023

Individual facility participation in LMIS stock reporting in the twelve-month period of October 2022–September 2023 was suboptimal (Fig. 2). Of the 1460 facilities in Sierra Leone, 1394 (95%) responded at any point during the 12-month period. Of the 1394 facilities that responded, 254 facilities (18.2%) met criteria for adequate reporting in all 12 months. 661 facilities (47.4%) met criteria for adequate reporting ten or more months, and 1173 facilities (84.1%) met criteria for adequate reporting seven months or more.

LMIS data analysis showed major shifts in national RDT distribution and the number of facilities reporting stockouts in May 2023, immediately preceding the national increase in confirmed malaria cases. Nationally, RDT distribution in May 2023 increased to 551,888 RDT test kits, a tenfold increase from the May 2019–2022 mean of 53,121, and 435% increase from the 2019–2022 monthly mean of 126,866 (Fig. 1c). This was the largest amount of RDTs delivered in a single month in Sierra Leone since electronic stock record began in 2019. The proportion of facilities reporting stockout during this period also changed significantly. In March 2023, 150 facilities (10.8%) reported stockout, whereas 35 (2.5%) did so in May 2023.

**Fig. 3:**
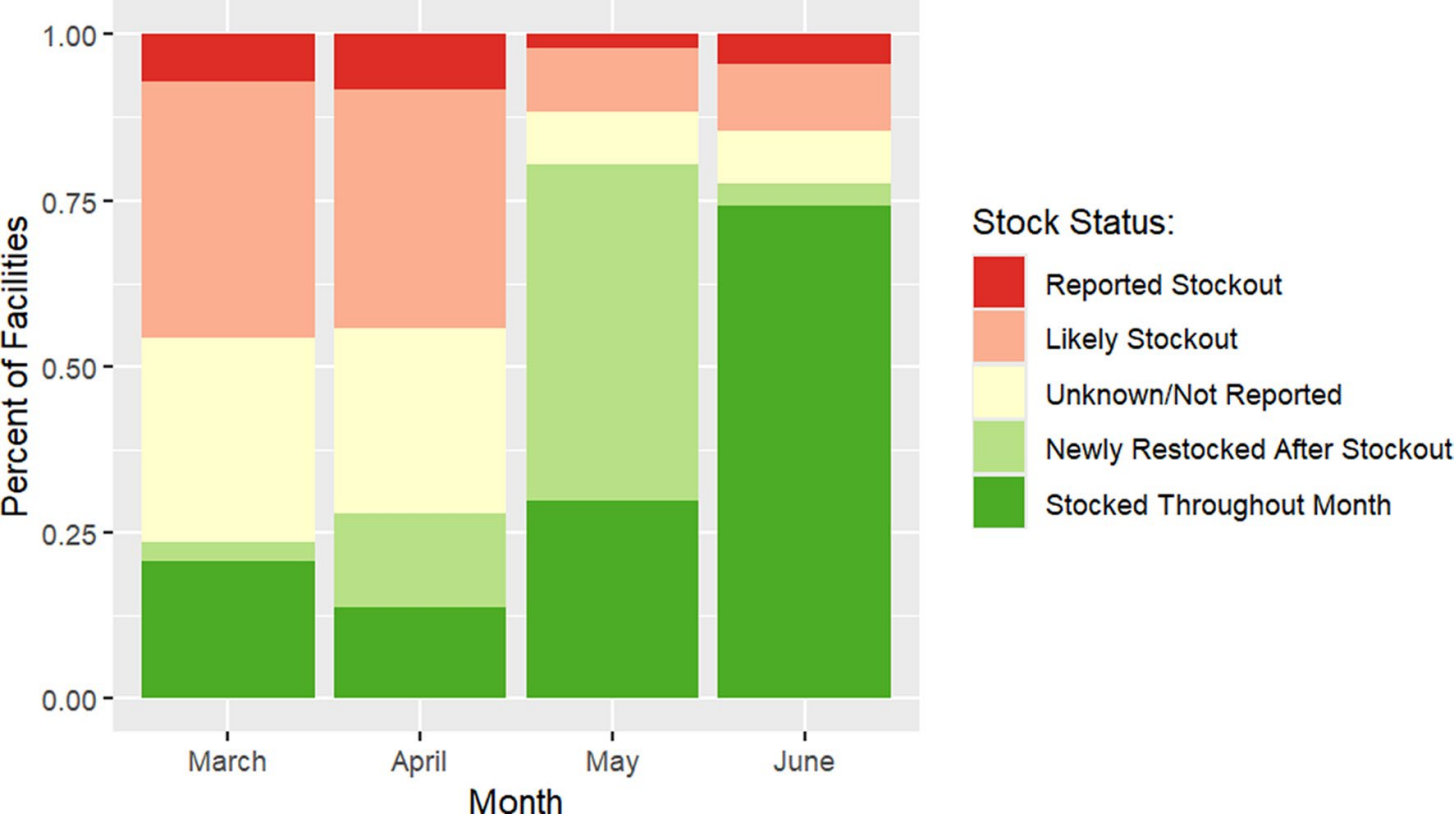
RDT Stock Status of Facilities by Month, Sierra Leone, 2023

The stock status analysis revealed that of the 1,394 facilities that reported stock data to LMIS during the October 2022-Septermber 2023 period, a small margin were stocked throughout the month in the months leading up to the spike in cases (Fig. 3 and Table 3). In March 2023, 288 facilities (20.7%) met criteria for “stocked throughout the month.” Only 191 facilities (13.7%) met such criteria in April. This changed to 1034 facilities (74%) meeting such criteria in June 2023, concurrent with the peak of confirmed malaria cases. A large proportion of facilities (707 facilities, 50.7%) met criteria for “newly restocked after stockout” in May, concurrent with the known large distribution of RDTs. There was an inverse trend for likely and reported stockouts. In March, 690 facilities (49.4%) met criteria for “likely or reported stockout,” and 642 facilities (46%) met such criteria in April. This number drastically decreased to 169 facilities (12%) in May and 209 (15%) in June. The proportion of “likely stockouts” was consistently larger than “reported stockouts.” Notably, the number of facilities that did not report or reported values that were uninterpretable decreased drastically from March (374 facilities, 26.8%) to May (104 facilities, 7.5%).

**Table 3:**
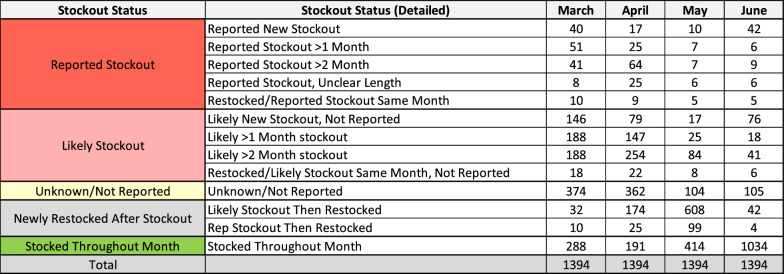
RDT Stock Status of Facilities by Month, Sierra Leone, 2023

## Discussion

This investigation was initially spurred by a rise in confirmed malaria cases reported nationally in routine HMIS surveillance data. Further exploration revealed that the elevation in cases was experienced by all age aggregations and geographic areas, with some regional variability in timing and magnitude. Clinical workers at the visited facilities discussed the regularity of RDT stockouts in their facilities, including the period before they were restocked and confirmed malaria surged at their facilities. The subsequent analysis on the national level revealed that the spike in confirmed cases occurred immediately after the largest distribution of RDT test kits in a single month on electronic record since 2019. As a result, about half of the country’s facilities, which were previously stockout out of RDTs, had the ability to test patients. The temporality of the spike in cases after a particularly large distribution of test kits suggested its cause to be increased access to and documented use of RDTs after prolonged stockouts.

Rapid diagnostic tests are a central aspect of malaria care management in Sierra Leone. National malaria case management guidelines direct clinicians to always get a parasitological diagnosis prior to treating with anti-malarials, and rapid diagnostic testing is the only option for community health workers and health facilities that are unable to perform microscopy [9]. At this point, guidelines do not provide clear direction for what clinical workers should do when a febrile patient presents for care and RDT’s are stocked out. It is not known how clinical workers respond during stockouts in Sierra Leone, though clinical workers sometimes treat empirically for malaria. However, previous research across Africa has demonstrated the deleterious impact facility stockouts: delayed malaria diagnosis/treatment, unnecessary anti-malarial use in patients with non-malarial febrile illnesses (NMFI), missed/delayed diagnosis of NMFI’s, reduced credibility of facilities/health workers in their communities, patient care seeking outside of the public system, reduced community case management, and potentially, increased antimalarial resistance [15–17]. In turn, delayed diagnosis of malaria has been demonstrated to increase risk of severe disease, and the out-of-pocket expenses of private care could lead to poor medication regimen adherence [18, 19].

Though it is well known that facilities in Sierra Leone experience stockouts of health commodities including malaria RDTs, it was unexpected that stockouts could lead to such pronounced aberrations in routine surveillance of confirmed cases, particularly on a national scale. However, the analysis showed that other potential causes, such as data recording errors or a true epidemic, were less likely. The 2023 spike in cases was experienced in all age groups and districts, making common data quality or entry errors an unlikely cause of the spike in cases. Though cases in Sierra Leone typically increase with the onset of the rainy season in April, the analysis showed that June 2023 was unlike any of the prior five Junes, making seasonal variation an unlikely aetiology. Moreover, the changes in calculated TPR were modest for a true outbreak. While there are no universally standardized TPR thresholds for declaring a malaria outbreak in highly endemic countries, it can be epidemiologically useful to monitor changes. Sierra Leone’s TPR in 2023 was higher than the 2018–2022 mean TPR for all twelve months, including months where there was not a contemporaneous rise in confirmed malaria, suggesting that other factors, such as testing rates, availability of tests, data reporting behaviours, and patient care-seeking, may have been at play.

Despite limited evidence supporting a true malaria outbreak, the temporal and geographic relationship of the spike in confirmed malaria cases and the large RDT distribution was compelling. Geographically, it is notable that three districts easily accessible to national health commodity storehouse in Freetown (Western Area Urban, Western Area Rural, Bo) peaked first, whereas a hard-to-reach district on the opposite end of the country (Kailahun) peaked last. Temporally, on a national level, only 191 of 1394 facilities (13.7%) were stocked throughout April, the month immediately before the large RDT distribution. This drastically changed the following month in June 2023, when confirmed malaria peaked in Sierra Leone. In June, 1034 of 1394 facilities (74%) were stocked throughout the month. Furthermore, the large proportion of facilities that met criteria for “newly restocked after stockout” in May (707 facilities, 50.7%), would make a rise in both testing and cases not only feasible, but rather likely. This change aligned with confirmed cases. Cases were below the 2018–2022 mean in March (− 4.2 σ) and April (− 6.8 σ), but rapidly increased in May (2.56 σ) and June (4.81 σ).

It is unclear why May 2023 was the largest distribution of RDTs on record, though it is possible that it was compensating for delayed distribution. Commodities in Sierra Leone are intended to be distributed from the national warehouse to the facilities on a quarterly basis. Prior to May 2023, the last major distribution of RDTs on the national level occurred in November 2022—six months before. Additionally, the preceding months had low distribution—40,076 kits in January 2023, 39,176 in February, and 24,814 in March. This was far eclipsed by the 550,388 kits distributed in May.

The results of this investigation revealed that stock reporting is suboptimal and RDT stockouts are likely more pervasive than reported in DHIS2. The analysis of facility participation in routine stock data revealed that only 18% of facilities reported for all 12 months assessed. The RDT stock status analysis revealed that of the likely stocked out facilities, the majority do not report their stockouts. Facilities with stock status “reported stockout” were consistently lesser in number than those with the status of “likely stockout.” This could potentially be due to facilities only reporting stockouts in the first month the stockout occurred and not in subsequent months of stockout, as was seen in the visited facilities.

The team wanted to verify the results of the stock status analysis against concrete survey data, and fortuitously there was a recent President’s Malaria Initiative supported End-Use Verification (EUV) survey on end-use health commodity stocks. The survey, which was conducted in September–October 2023 and randomly sampled 72 facilities in 12/16 districts, reported an RDT stockout rate of 23% on the day of visit, approximately double the rate reported in routine HMIS stock data during the same period [20]. In LMIS, 134 of 1085 (12.3%) responding facilities reported RDT stockouts in September, whereas 109 of 1000 (10.9%) responding facilities reported RDT stockout in October. When the parameters of this investigation’s RDT stock status analysis were applied to this period, it showed a stockout rate of 29.2% in September and 29.3% in October. The closeness of the reported EUV findings to that of this investigation’s stock status analysis verified that it is a dependable measure of stock status. Furthermore, it provided concrete evidence that RDT stockout rates may be higher than reported in routine LMIS data.

This investigation had several limitations. Site visits were few due to time and resource limitations– just four facilities known to have substantial spikes in malaria cases in two districts. Though the site visits were not the central evidence in this investigation, the staff interviews were invaluable, prompting a national analysis of facility stocks. A second limitation was the inability to calculate a true test positivity rate. The team made efforts to mitigate bias in TPR calculation, but the true national TPR was unknown because of differential reporting biases, such as facilities’ inclination to report positive test results more frequently than negative results. A third limitation was the relative paucity of electronic stock data in LMIS. LMIS stock data in DHIS2 were only available from 2019 onward, and thus the comparison was limited to four years instead of the ideal five. Lastly, the stock status analysis had limitations. The various stock statuses were designed using logical criteria to draw more information from the existing imperfect data. As such, they were limited by missing data. Most facilities met criteria for one of the 12 stock statuses, ranging from 1020 of 1394 facilities (73%) in March to 1290 (92.5%) in June. This enabled us to infer stock status about many of the facilities in Sierra Leone. A significant number of facilities, however, did not report or did not meet logical criteria, and thus were classified as “unknown/not reported.”

Interestingly, the number of facilities classified as “unknown/not reported” decreased from March (374 facilities, 26.8%) to May (104 facilities, 7.5%) without additional training in this period. The shift suggests that these facilities may have been stocked out or otherwise impacted by the large RDT distribution as well. The situation in these facilities remains a mystery, and determining their true status would likely require in-person analysis of non-digitized facility registers.

This analysis showed that prolonged RDT stockouts can cause pronounced aberrations in routine national malaria surveillance of confirmed cases. During such stockouts, large portions of the country are unable to test and report confirmed cases. In this way, confirmed case surveillance become representative not of the country, but rather the portion able to test, and true variations in disease incidence would be masked. In turn, when a large portion of facilities suddenly have renewed ability to test and report upon being restocked with RDTs, large increases in confirmed cases mimic true outbreaks, triggering response activities like ours. In this way, prolonged stockouts can complicate ability to recognize true variations in malaria cases, and thus provide effective surveillance.

In light of these findings, Sierra Leone and its partners could strengthen supply chain logistics and health commodity stock tracking to ensure consistent supply of RDTs and more robust malaria surveillance data. The results of this investigation support the need to improve the consistency of stock reporting, particularly in the event of a stockout and continuously until the facility is restocked, to decrease stockout frequency and duration. Thus, facility level barriers to more complete and consistent reporting of stock data can be identified so that targeted interventions could be instituted as appropriate. This investigation also highlights the potential for Sierra Leone and its partners to utilize routine LMIS data to improve health commodity stock tracking nationally. Alternative indicators, such as proportion of facilities that both begin and end each month with a positive balance, could be utilized to monitor stock statuses. Though this investigation presents some areas to improve health commodity supply, additional research is needed to identify recurrent obstacles and pitfalls at all levels of the health commodity supply chain.

In conclusion, the May–July 2023 spike in malaria cases was likely related to increased access to and use of testing immediately following a period of limited RDT stocks. This situation underscores how fluctuations in RDT availability and use complicate ability to recognize true variations in malaria cases, and thus provide effective surveillance. Commodity stockouts were underrepresented in routine reporting, potentially due to suboptimal facility participation in LMIS stock reporting or improper reporting of stockouts. Sierra Leone and partners can strengthen supply chain logistics and health commodity stock tracking to ensure consistent supply of RDTs and more robust malaria surveillance data.

## Data Availability

Data are available upon reasonable request to the authors.
